# Identification, Classification, and Transcriptional Analysis of *Rab GTPase* Genes from Tomato (*Solanum lycopersicum*) Reveals Salt Stress Response Genes

**DOI:** 10.3390/genes15040453

**Published:** 2024-04-03

**Authors:** Flavia Soto, Alex San Martín-Davison, Josselyn Salinas-Cornejo, José Madrid-Espinoza, Simón Ruiz-Lara

**Affiliations:** Laboratorio de Genómica Funcional, Instituto de Ciencias Biológicas, Universidad de Talca, Talca 3460000, Chile; flsoto@utalca.cl (F.S.); alexsanmartin@utalca.cl (A.S.M.-D.); josalinas@utalca.cl (J.S.-C.); jmadrid@utalca.cl (J.M.-E.)

**Keywords:** Rab GTPases, genome wide identification, vesicular trafficking, expression profiles, salt stress response, tomato

## Abstract

Salinity in plants generates an osmotic and ionic imbalance inside cells that compromises the viability of the plant. Rab GTPases, the largest family within the small GTPase superfamily, play pivotal roles as regulators of vesicular trafficking in plants, including the economically important and globally cultivated tomato (*Solanum lycopersicum*). Despite their significance, the specific involvement of these small GTPases in tomato vesicular trafficking and their role under saline stress remains poorly understood. In this work, we identified and classified 54 genes encoding Rab GTPases in cultivated tomato, elucidating their genomic distribution and structural characteristics. We conducted an analysis of duplication events within the *S. lycopersicum* genome, as well as an examination of gene structure and conserved motifs. In addition, we investigated the transcriptional profiles for these *Rab GTPases* in various tissues of cultivated and wild tomato species using microarray-based analysis. The results showed predominantly low expression in most of the genes in both leaves and vegetative meristem, contrasting with notably high expression levels observed in seedling roots. Also, a greater increase in gene expression in shoots from salt-tolerant wild tomato species was observed under normal conditions when comparing *Solanum habrochaites*, *Solanum pennellii*, and *Solanum pimpinellifolium* with *S. lycopersicum*. Furthermore, an expression analysis of *Rab GTPases* from *Solanum chilense* in leaves and roots under salt stress treatment were also carried out for their characterization. These findings revealed that specific Rab GTPases from the endocytic pathway and the trans-Golgi network (TGN) showed higher induction in plants exposed to saline stress conditions. Likewise, disparities in gene expression were observed both among members of the same Rab GTPase subfamily and between different subfamilies. Overall, this work emphasizes the high degree of conservation of Rab GTPases, their high functional diversification in higher plants, and the essential role in mediating salt stress tolerance and suggests their potential for further exploration of vesicular trafficking mechanisms in response to abiotic stress conditions.

## 1. Introduction

Salt stress has a detrimental effect on plants, limiting their development and reproduction. Nearly 4.12 million km^2^ are affected by excessive salinity, equivalent to 6% of the global arable land, a problem that has been intensified by human activity and climate change [1]. It is estimated that 25% of arable land will be affected in the next 25 years, while by the end of the 21st century, South America, southern Australia, Mexico, south-west United States, and South Africa will be at risk of higher soil salinity caused by climate change [2]. In this context, it is important to find and characterize the determinants of the tolerance capacity of plants that allow the adverse effects of salinity to be avoided, as well as physiological or molecular mechanisms associated with the response and its adaptation to salt stress [3].

The tomato (*Solanum lycopersicum*) is a main crop, with great economic relevance and the largest global cultivation extent. Native to South America, its fruit is highly prized in world markets for both fresh or processed consumption. Also, it is recognized as a model species in genetics and research associated with different studies, including stress tolerance mechanisms [4]. Its moderately sensitive tolerance capacity to salt stress classifies it as a glycophyte species. At a physiological level, salt stress leads to decreased germination capacity, reduced osmotic potential, and diminished development of roots, leaves, flowers, and fruits due to inhibition of cell division and elongation [5]. At the biochemical level, salt-induced overproduction of reactive oxygen species (ROS) results in damage to key cellular components, such as proteins, lipids, and nucleic acids [5]. To counteract these adverse effects, the production of compatible osmolytes and the activation of the antioxidant system are essential to maintain cellular integrity. Significant progress has been made in understanding more sophisticated subcellular mechanisms of tolerance in tomato plants. Recent research has revealed that cellular damage repair systems and ion compartmentalization, based on vesicle trafficking, play a fundamental role in adaptation to saline conditions [6,7,8]. These emerging mechanisms provide a more comprehensive understanding of how plants respond and adapt to saline environments, opening new perspectives for genetic improvement strategies and agricultural practices that promote stress tolerance in tomato crops.

Intracellular vesicular trafficking is directed by a family of proteins known as Rab GTPases. These constitute the largest family within the superfamily of small GTPases involved in the formation, transport, docking, and fusion of vesicles. Functioning as molecular switches, Rab GTPases cycle between the “active” and “inactive” state, a dynamic process linked to the binding and hydrolysis of GTP. This mechanism involves collaboration with other proteins such as GEF, GAP, and GDI [9,10,11,12].

Under salt stress conditions, response mechanisms are activated that seek to protect and change damaged structures. This requires the removal of damaged molecules in different cellular compartments and their subsequent replacement by new ones. Transport to and from specific compartments for recycling and elimination of macromolecules occurs through an intracellular vesicular trafficking system. While vesicular trafficking has conventionally been viewed as a fundamental process in both animals and plants; primarily involved in the transport and renewal of cellular structures, there is mounting evidence suggesting its pivotal role in stress adaptation [13,14,15,16,17,18]. For instance, research has demonstrated that a mutation in RabF2b of the endocytic pathway enhances salt stress tolerance [14], while RabA1 from the TGN and PM pathway is essential for salinity tolerance in *Arabidopsis thaliana* [15].

The role of vesicular traffic in maintaining ionic homeostasis includes the following key functions: (1) releasing transporters attached to the tonoplast or plasma membrane, relocating channels, and facilitating ATP-driven pumps directly involved in the ion transport process [19]; (2) transporting of cargo proteins to specific organelles [20]; and (3) recycling stress signals [21] or replacing other membrane components [22] that mediate in osmotic stress responses. Therefore, an increase in the efficiency of protein trafficking technology, such as anchoring, fusion, and recycling, is expected to positively impact plant adaptations to high-salinity environments.

In vivo and in vitro experiments have demonstrated the role of this class of proteins in intracellular membrane trafficking [10,15]. ARA6 modulates the assembly of a SNARE complex distinct from the conventional RAB5 and has a functional role in response to salinity stress in *A. thaliana*. In plants, there is a unique trafficking pathway mediated by this Rab GTPase [23,24]. ARA6 (AtRabF1) was subsequently shown to be involved in salt stress tolerance and dark-induced senescence [25]. Another gene of this family, Rab5, was studied in mango (*Mangifera indica*), where it was found that *MiRab5* expression increased during the later stages of fruit ripening. In addition, *MiRab5* was generally up-regulated in response to various abiotic stresses (cold, salinity, and PEG treatments) [26]. In *A. thaliana*, members of the RabA (RabA1a-d) subfamily, involved in the Trans Golgi Network (TGN), are required for salt stress tolerance, and their function has been described as redundant [15]. Overexpression of the *Rab7* gene from *Pennisetum glaucum*, a relatively abiotic stress-tolerant species, enhanced NaCl tolerance in transgenic tobacco [27].

Additionally, given the specific distribution of Rab GTPases in the different cell membranes, it was hypothesized that Rab GTPases, along with SNARE proteins, provide specificity for membrane fusion events [10]. Vernoud et al. [28] identified 57 isoforms of Rab GTPases, which they named AtRab GTPases, and grouped them into eight subfamilies (AtRabA–AtRAbH) according to the subfamilies found in other organisms such as humans and yeast [29].

For this work, we identified 54 Rab GTPases from *S. lycopersicum*, classified them into eight subfamilies, and analyzed their transcriptional profiles in cultivated and wild tomato species. Additionally, we determined the expression profile of 10 representative *Rab GTPases* genes of each subfamily in the halophyte species *Solanum chilense* under salt stress conditions. Altogether, this evidence suggests the potential utility of Rab GTPases from *S. chilense* for future overexpression studies in *S. lycopersicum* to improve its tolerance to abiotic stress.

## 2. Materials and Methods

### 2.1. Identification of Rab GTPases in the S. lycopersicum Genome

*A. thaliana* Rab GTPase sequences obtained in the TAIR database (http://www.arabidopsis.org/, accessed on 29 February 2024) were used to search the genome of *S. lycopersicum* using the BLAST program integrated into the SolGenomics website http://solgenomics.net/tools/blast/index.pl (accessed on 10 October 2023). Of the sequences obtained, the duplicates were eliminated and analyzed in search of recognizable domains using the SMART site http://smart.embl-heidelberg.de/ (accessed on 20 October 2023), based on HMMER and the InterPro website https://www.ebi.ac.uk/interpro/ (accessed on 30 October 2023). For each Rab sequence found in the *S. lycopersicum* genome, the chromosome to which it belongs, the start and end positions in the genome, the strand in which it is found, and the size of the protein were determined. The isoelectric point (pI) and molecular weight (MW) were predicted using the Expasy Compute pI/Mw tool http://web.expasy.org/compute_pi/ (accessed on 10 November 2023). Values of hydropathy (GRAVY value) were calculated using the Expasy ProtParam tool http://expasy.org/tools/protparam.html (accessed on 14 November 2023). In addition, the subcellular locations were determined using CELLO V2.5 http://cello.life.nctu.edu.tw (accessed on 24 November 2023) and Wolf PSORT https://wolfpsort.hgc.jp (accessed on 26 November 2023) tools. For phylogenetic analysis, the sequences obtained were aligned with ClustalO https://www.ebi.ac.uk/jdispatcher/msa/clustalo (accessed on 28 November 2023), and subsequently, the MEGA 7 program https://www.megasoftware.net/ (accessed on 29 November 2023) was used for the construction of the tree using the Neighbor-Joining method and 1000 bootstrap replications.

### 2.2. Structure of Rab Genes and Conserved Motifs in Rab Proteins

Intron–exon distribution of putative Rab GTPases from *S. lycopersicum* was illustrated using the Gene Structure Display Server (GSDS) http://gsds.gao-lab.org/ (accessed on 14 October 2023), using the information available for each sequence in SolGenomics. Web LoGo https://weblogo.berkeley.edu/ (accessed on 17 October 2023) was used for the comparison of conserved amino acid sequences. From the result obtained, the presence of the RabF, RabSF, and G motifs, which are characteristic of Rab GTPases, was determined.

### 2.3. Rab GTPase Expression Profiles in Databases

Expression profiles of different tissues were obtained from the Bio-Analytic Resource Database http://bar.utoronto.ca (accessed on 21 October 2023). For *S. lycopersicum* and *Solanum pennellii*, the following tissues were considered: flower, stem, leaf, vegetative meristem, seedling shoot, seedling root, mature fruit, and developing fruit [30]. The expression values obtained were normalized with the control values from the database.

Additionally, expression profiles of Rab GTPases for four tomato species (*S. lycopersicum*, *Solanum pimpinellifolium*, *Solanum habrochaites*, and *S. pennellii*) were obtained from the “Tomato Expression” database to compare *S. lycopersicum* with its salt-tolerant wild relatives. Values were normalized to *S. lycopersicum* [30].

### 2.4. Plant Material and Growth Conditions

Seeds of *S. chilense* (Dunal) were collected from plants in Northern Chile at a 2500 m.a.s.l., 18° 26′ S lat. 69° 45′ long (date of collection: March 2019). Clonal plants were obtained by propagation in pots, which contained a mixture of perlite, vermiculite, and peat moss (1:1:1 *v*/*v*) and were grown under greenhouse conditions. The conditions used were 23–25 °C and a 16 h/8 h light/dark photoperiod. To fertilize plants, commercial Hoagland’s solution (1/4 strength) every 10 days was used. The salt stress assay was performed as follows: 7-week-old *S. chilense* plants were grown in 2-litre pots containing a perlite:vermiculite mixture (1:1 *v*/*v*) and irrigated with 400 mL of Hoagland’s solution containing 300 mM NaCl. Leaf and root samples were collected at 0, 3, 6, 12, 24, 48 and 72 h after salt treatment and were immediately frozen with liquid nitrogen and stored at −80 °C.

### 2.5. RNA Extraction and cDNA Synthesis

Total RNA was extracted from 100 mg of *S. chilense* leaves and roots using the SV Total RNA Isolation System kit (Promega, Madison, WI, USA) following the protocol indicated by the manufacturer. A total of 80 μL of the final volume was obtained from each sample and treated with TURBO DNA-free™ Kit (Invitrogen, Carlsbad, CA, USA) to eliminate genomic DNA contamination. The RNA concentration was quantified at 260/280 nm in an Infinite^®^ 200 PRO NanoQuant (Tecan Group Ltd. Männedorf, Switzerland) spectrophotometer. The samples were stored at −20 °C.

The reverse transcription reaction for the synthesis of the first strand of cDNA was performed using 2 μg of total RNA treated with DNAse, extracted from leaf and root samples. For these reactions, the First Strand cDNA Synthesis Kit (Thermo, Carlsbad, CA, USA) was used, under the general conditions described by the provider. The mix for each reaction contained 4 μL 5X Reaction buffer, 1 μL Ribolock RNAse inhibitor, 2 μL 10 mM dNTP Mix, 2 μL M-MuLV Reverse Transcriptase, 1 μL Oligo(dT), and a mix of 10 μL RNA + H_2_0 according to the concentration to be used, to make a total volume of 20 μL. The synthesis was conducted by incubating the samples at 37 °C for 60 min and a final incubation time at 70 °C for 5 min to stop the reaction. The cDNAs were stored at −20 °C.

### 2.6. Analysis of Gene Expression

Analysis of the expression of the genes of interest was determined by real-time PCR (qRT-PCR) using an Mx3000P qPCR System thermal cycler (Stratagene, La Jolla, CA, USA). cDNA amplification reactions were conducted using the Maximum SYBR Green/ROX qPCR Master Mix (2X) method (ThermoScientific, MA, USA) in a final volume of 20 μL and according to the manufacturer’s recommendations. Each 20 μL reaction consisted of 2 μL of diluted cDNA (50 ng), 10 μL of 2X Maxima^®^ SYBR Green/ROX qPCR Master Mix reagent, 0.5 μL of each primer at a concentration of 0.25 μM, and nuclease-free water. Three determinations were made for each of the three biological replicates, and a negative control was also included. The temperature settings used were as follows: 95 °C for 10 min, followed by 40 cycles of 95 °C for 15 s, 58–60 °C for 15 s, and 72 °C for 20 s. At the end of each amplification step, the fluorescence was measured and at the end of amplification; a denaturation curve was established by continuously reading the fluorescence during a temperature increase from 55 °C to 95 °C.

Gene expression was normalized based on the relative expression values of the gene that codes for *Ubiquitin3* in *S. lycopersicum* (*SlUBI3*) [31]. For the candidate genes, specific primers were designed from their UTR-3’ region with the Beacon Designer 4 program. The nucleotide sequence of the primers analyzed by qRT–PCR are described in Table S1. The data obtained at the end of each run were analyzed manually, and the calculations to estimate the relative expression of each gene were determined following the method described by Pfaffl [32].

### 2.7. Statistical Analysis

Statistics were performed in R (version 1.7). Statistical significance was analyzed by one-way ANOVA followed by the post hoc Tukey HSD test (*p* < 0.05).

## 3. Results

### 3.1. Identification and Phylogenetic Analysis of Rab GTPase Genes in S. lycopersicum

The 57 known sequences of Rab GTPases from *A. thaliana* [28] were utilized to conduct a search in the *S. lycopersicum* genome using the BLAST program integrated into the SolGenomics Database. Of the obtained sequences, duplicates were eliminated, and the remaining sequences were analyzed to identify recognizable domains. A total of 54 sequences were found in the *S. lycopersicum* genome (Table 1).

**Figure 1:**
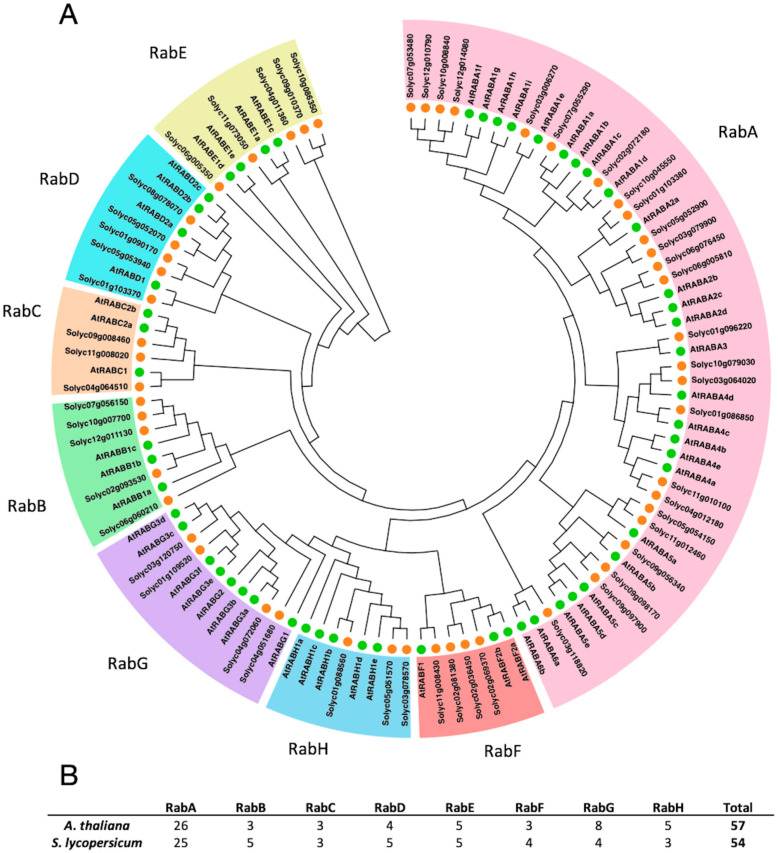
Identification and distribution of Rab GTPases in the genome of *Solanum lycopersicum*. In order to classify the putative Rab GTPases of *S. lycopersicum*, a search of the *Arabidopsis thaliana* Rab GTPase sequences obtained from the TAIR database was performed. (**A**) Phylogenetic tree constructed using the Neighbor-Joining method with 1000 bootstrap replicates. Rab GTPases from *A. thaliana* are represented by a green circle, and those from *S. lycopersicum* by an orange circle. (**B**) Table showing the number of Rab GTPases in *A. thaliana* and *S. lycopersicum*, as well as the number per subfamily in these species.

For the phylogenetic analysis, full-length sequences of 57 *A. thaliana* and 54 *S. lycopersicum* Rab GTPases were aligned, facilitating the construction of a comprehensive phylogenetic tree. Notably, all sequences from *S. lycopersicum* were included in the clades of the eight subfamilies of Rab GTPases (RabA–RabH) (Figure 1). In detail, the *S. lycopersicum* clade A encompasses 25 members, while *A. thaliana* hosts 26 members; clade B harbors 5 members in tomato and 3 in *A. thaliana*; clade C contains 3 members in both species; clade D comprises 5 members in *S. lycopersicum* and 4 in *A. thaliana*; clade E exhibits 5 members in *S. lycopersicum* and 4 in *A. thaliana*; clade F consists of 4 members in *S. lycopersicum* and 3 in *A. thaliana*; clade G encompasses 4 members in *S. lycopersicum* and 8 in *A. thaliana*; and finally, clade H comprises 3 members in *S. lycopersicum* and 5 in *A. thaliana*.

### 3.2. Duplication between Rab Genes from S. lycopersicum

**Figure 2:**
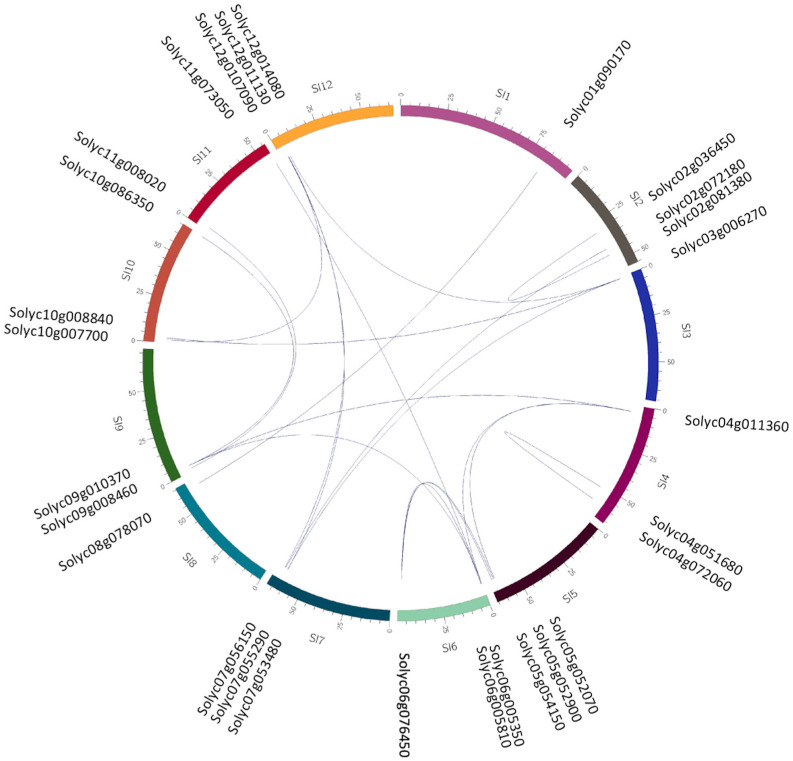
Genome-wide duplication of *Rab GTPases*. Chromosome locations and duplication of *Solanum lycopersicum Rab GTPases*. Chromosomes are displayed in different colors. Duplicated *Rab GTPases* are labeled and linked with blue lines. Duplication analysis between Rab genes was carried out using the chromosomal locations of Rab GTPases obtained from the PGDD database http://chibba.agtec.uga.edu/duplication/ (accessed on 3 November 2023). The graphic representation was made using the web-based service ClicO FS, freely available at http://clicofs.codoncloud.com (accessed on 15 December 2023).

The presence of duplication events within the *S. lycopersicum* genome was assessed. These events are represented in Figure 2 as blue lines, indicating 28 duplication events within the genome. A total of 54 *Rab GTPases* of *S. lycopersicum* were distributed across the 12 chromosomes (Figure 1B), with chromosome 8 having the lowest number of *Rab GTPases*, with only one. Chromosomes 7 and 12 contain three *Rab GTPases* each. Of the total, six chromosomes have five Rabs each (2, 4, 5, 9, 10, and 11). Chromosome 3 has six *Rab GTPases*, chromosome 4 has four *Rab GTPases,* and finally, the chromosome with the highest number is chromosome 1, with seven *Rab GTPases*.

### 3.3. Structure of Rab Genes and Conserved Motifs of Rab Proteins in S. lycopersicum

**Figure 3:**
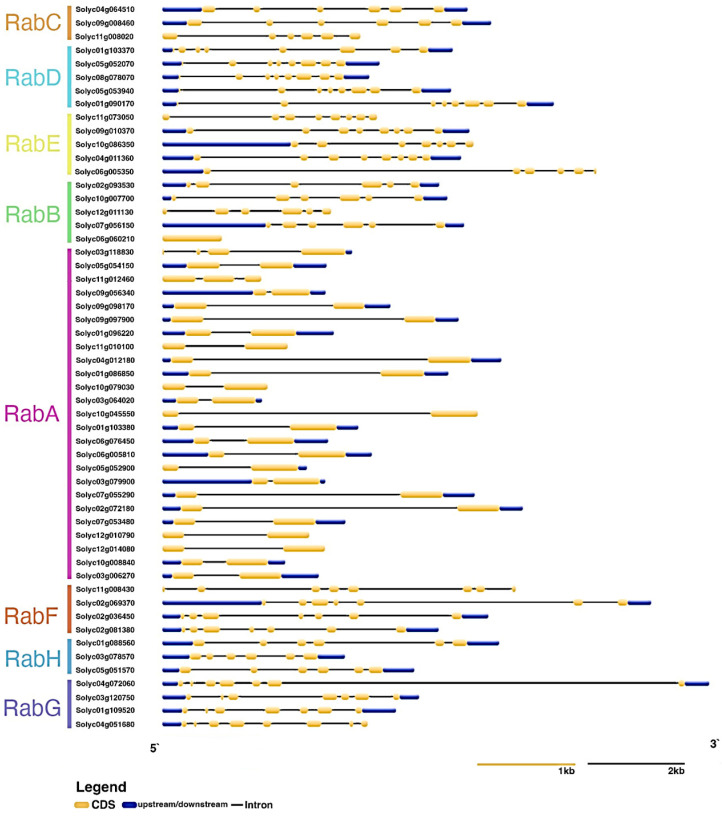
Gene structure of *Rab GTPases* from *Solanum lycopersicum*. Intron–exon distribution according to the RabA–RabH subfamilies. In yellow, CDS; in blue, upstream/downstream regions; in black, introns.

The intron–exon distribution of the *Rab GTPase* genes of *S. lycopersicum* was analyzed (Figure 3). Genes were grouped according to their respective subfamilies (RabA–RabH). Members of the RabB, RabC, and RabH subfamilies have six exons; one member of the RabB subfamily lacks introns entirely. Conversely, members of the RabD, RabE, RabF, and RabG subfamilies possess between seven and eight exons. Notably, the RabA subfamily stands out with the fewest introns, predominantly featuring only one intron per gene.

**Figure 4:**
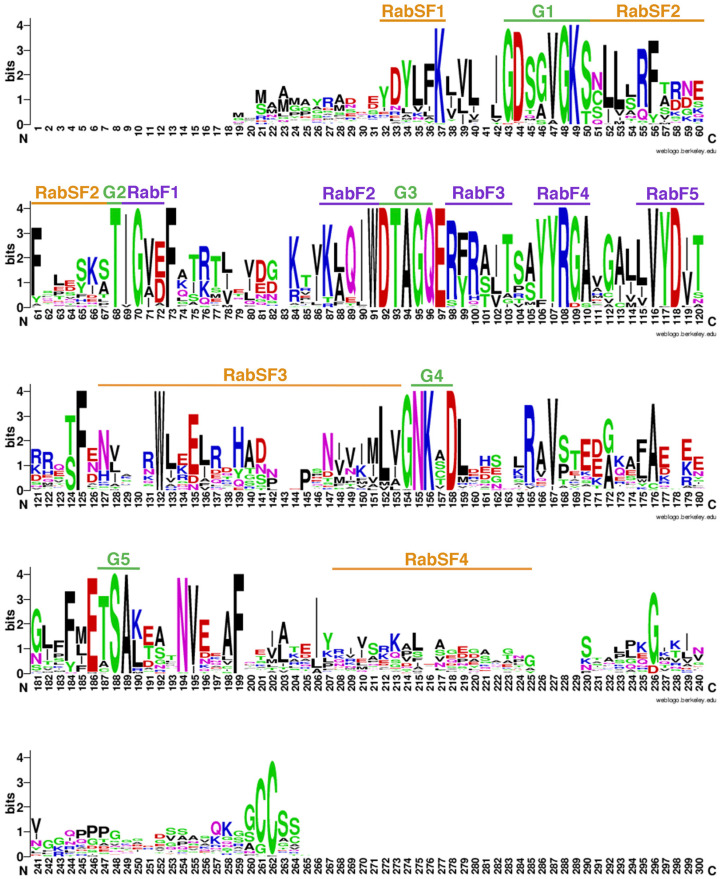
Sequence logo comparison of the 54 Rab GTPase proteins of *Solanum lycopersicum*. RabSF motifs are indicated in orange, RabF motifs in purple, and G motifs in green.

The alignment of 54 putative Rab GTPase protein sequences from *S. lycopersicum* was conducted to identify conserved motifs (Figure 4). Remarkably, the presence of five RabF motifs (RabF1–RabF5) (Figure 4, marked in purple) stands out; these are hallmark features of Rab GTPases, conferring specificity towards other Rab-interacting proteins like REP and GDI [33]. Additionally, RabSF motifs specific to each Rab GTPase subfamily (RabA–RabH) were identified. These motifs play a crucial role in facilitating binding between Rab and its respective effectors [33]. In addition, residues corresponding to the hydrophobic triad (F, W, and Y) were identified as highly conserved and essential for effector recognition [34,35]. Structures essential for the interaction with GDI and other proteins, including Switch I, Interswitch, and Switch II, were found, emphasizing their importance in the Rab GTPase system [36].

### 3.4. Expression of Rab GTPases in Different Tissues of Cultivated and Wild Tomato

**Figure 5:**
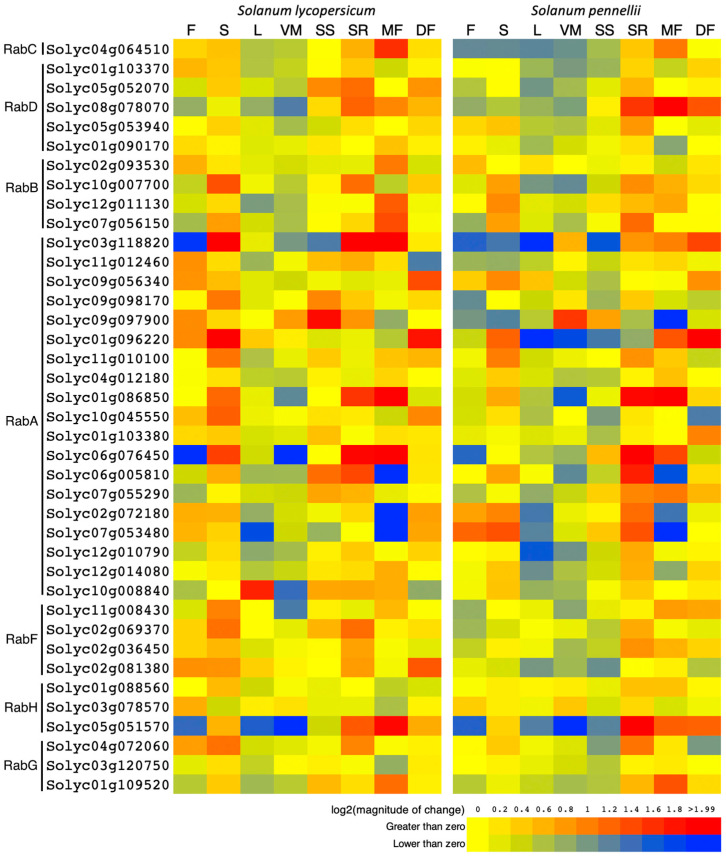
Expression patterns of *Rab GTPase* genes in different tissues, organs, and developmental stages of tomato. Positive and negative gene expression values are shown in red and blue, respectively. F: flowers; S: shoots; L: leaves; VM: vegetative meristems; SS: seedling shoots; SR: seedling roots; MF: mature fruit; DF: developing fruit.

To determine the expression profile in different tissues, two tomato species were analyzed: *S. lycopersicum*, a cultivated species, and its wild relative, *S. pennellii*. The expression patterns in flower, stem, leaf, vegetative meristem, seedling shoot, seedling root, mature fruit, and developing fruit are presented in Figure 5 for both species. Overall, it is observed that expression levels in leaves and vegetative meristem tend to be negative or close to zero, with few exceptions, such as Solyc10g008840 for leaf in *S. lycopersicum* and Solyc09g097900 in the vegetative meristem. On the other hand, significant positive expression values are detected in seedling roots, although there are instances of slightly negative expression, as seen in Solyc01g096220, corresponding to a Rab GTPase of the subfamily A. Interestingly, there is no discernible pattern in terms of expression associated with a particular species or subfamily; instead, expression varies among individual Rab GTPases.

**Figure 6:**
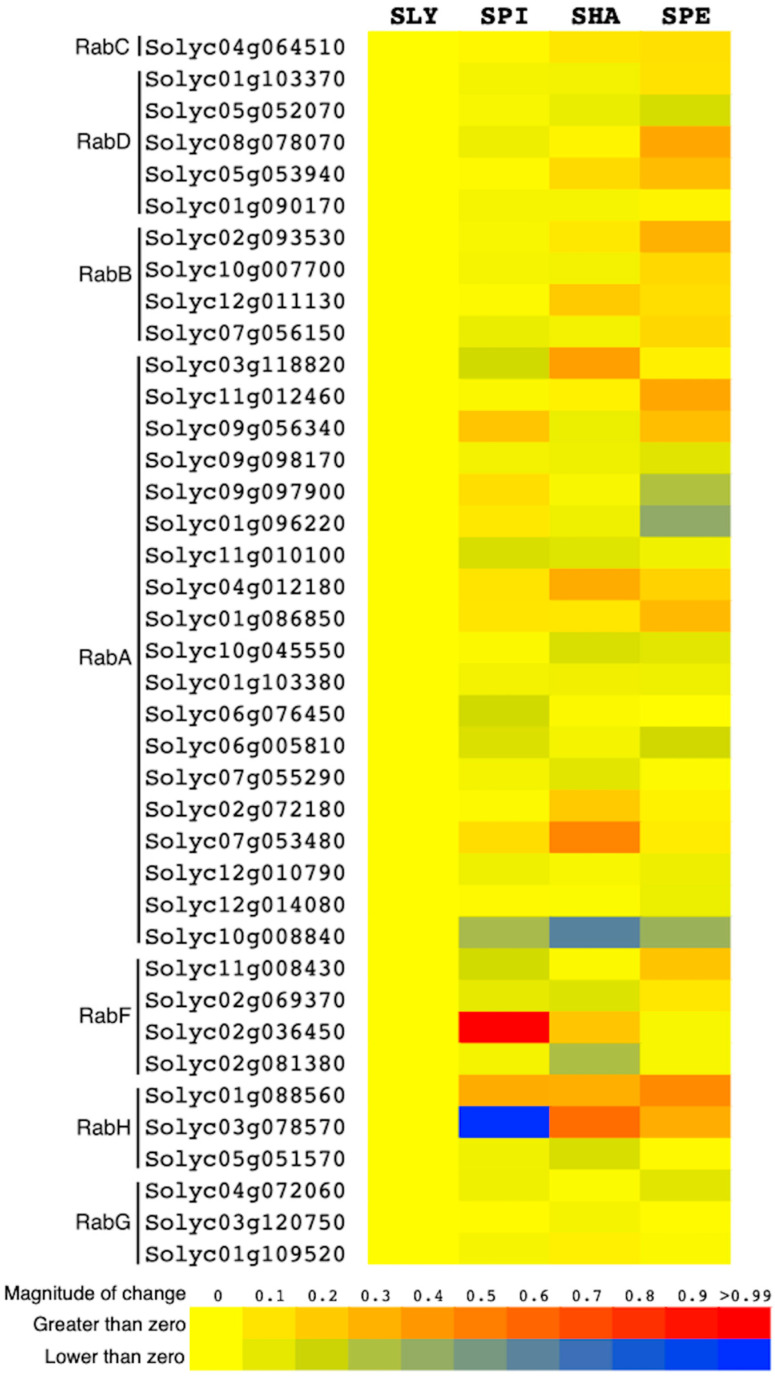
Expression profile of *Rab GTPase* genes in four tomato species under normal conditions. Expression values were obtained from the “Tomato Expression” database (http://malooflab.phytonetworks.org/apps/tomato-expression/ accessed on 21 October 2023). Transcript levels of *Rab GTPase* genes from wild tomato species relative to *S. lycopersicum* are represented. Positive and negative gene expression values are shown in red and blue, respectively. Nomenclature: SLY: *Solanum lycopersicum*; SHA: *Solanum habrochaites*; SPE: *Solanum pennellii*; and SPI: *Solanum pimpinellifolium*.

Furthermore, with the purpose of studying the expression profile of *Rab GTPases* in salt-tolerant tomato species, analysis was conducted for *S. lycopersicum* and three wild relatives: *S. pimpinellifolium*, *S. habrochaites*, and *S. pennellii* (Figure 6). Expression values are predominantly positive for most of the *Rab GTPases* in the wild species compared to *S. lycopersicum*. In addition, significant differences are observed in the transcriptional response of the genes among the different wild species, suggesting a differential regulation of expression of the same genes in the different species. Examples of this include Solyc06g076450, which exhibits the highest expression value among *Rab GTPases* in *S. pimpinellifolium* and has a similar value in both *S. lycopersicum* and *S. pennellii*. A similar situation occurs with Solyc03g078570, which shows the lowest expression value in *S. pimpinellifolium* and positive expression in *S. pennellii* and *S. habrochaites*.

### 3.5. Differential Expression of Rab GTPase Genes from S. chilense in Leaves and Roots under Saline Stress

In order to have a better approximation regarding the transcriptional profile of Rab GTPases under salt stress conditions in wild species, representatives of each family of Rab GTPases were selected from the salinity-tolerant species *S. chilense*. Due to the large number of genes encoding Rab GTPases found in the genome of *S. lycopersicum* and considering that many of these genes perform redundant functions, the selection of genes was based on two criteria: the size of the subfamily and the vesicular trafficking pathway in which they are fulfilling their function. Therefore, we selected putative homologous members of the endocytic pathway, such as SlRabF1 and SlRabG3e; members of the secretory pathway, such as SlRabB1b, and the retrograde pathway (Golgi complex–Rough Endoplasmic Reticulum), such as SlRabH1b; and members of the TGN as part of the RabA subfamily. The expression levels of these genes in *S. chilense* under salt stress conditions were determined using their homologues. The Rab GTPases analyzed are listed in Table 2.

Seven-week-old *S. chilense* plants were subjected to salt stress through single irrigation with 400 mL of Hoagland’s solution containing 300 mM NaCl, followed by regular watering. Physiological parameters were measured, and a molecular stress marker gene, *TSW12*, was utilized to confirm the response to salt stress in these plants (Figure S1) [37].

**Figure 7:**
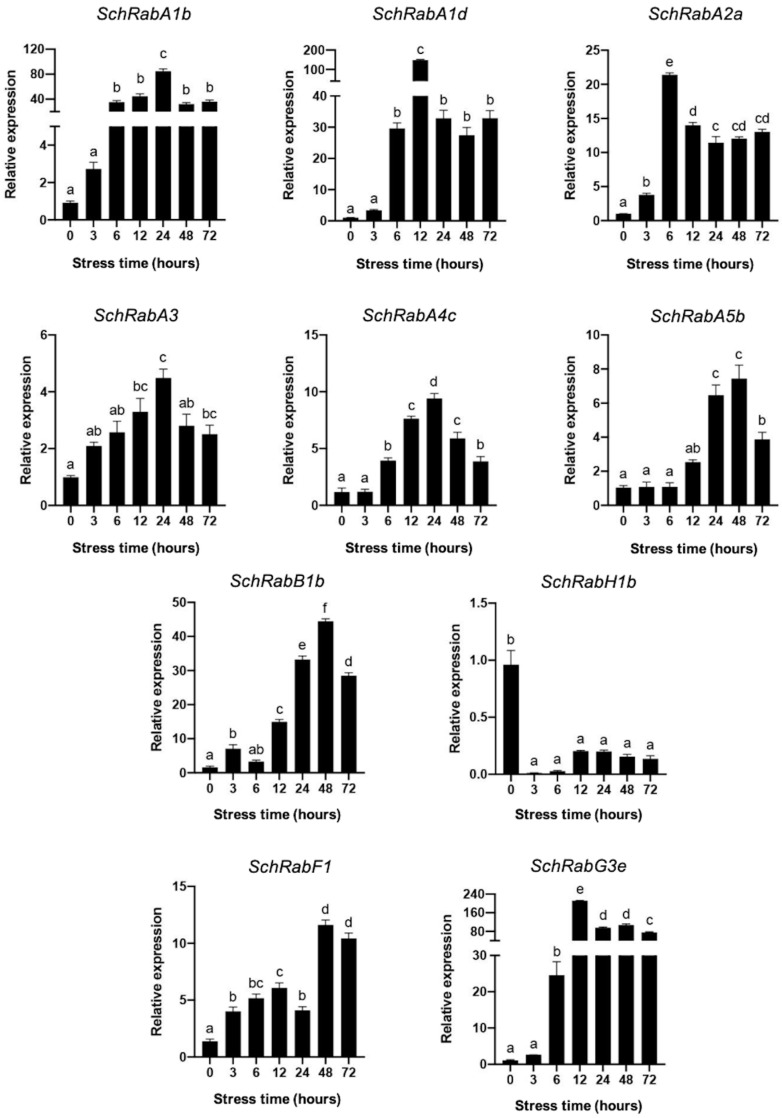
Relative expression of *Rab GTPases* under salt stress in *Solanum chilense*. Relative expression of RabA subfamily members from the TGN trafficking pathway, with *SchRabB1b* belonging to the secretory pathway, *SchRabH1b* to the retrograde route, and *SchRabF1* and *SchRabG3e* to the endocytic pathway. Black bars indicate the relative expression of *Rab GTPase* genes in leaves. Different letters indicate significant differences (*p* < 0.05) according to a one-way ANOVA test.

Subsequently, the transcript levels of the different *Rab GTPases* were evaluated in leaves of the *S. chilense* plants under salt stress conditions (Figure 7). Of the genes evaluated, all except *SchRabH1b* exhibited an increase in transcript levels as the experiment progressed. Notably, there are differences in salt-induced expression levels between members of the same subfamily, as observed in representatives of the *SchRabA* subfamily, as well as between subfamilies. For instance, *SchRabA1b*, *SchRabA1d*, and *SchRabG3e* displayed a significant increase in their relative expression from 3 h onwards. Moreover, differences were also observed in the induction times of each gene. Specifically, *SchRabA5b* appeared to only induce its expression 12 h after the onset of stress.

**Figure 8:**
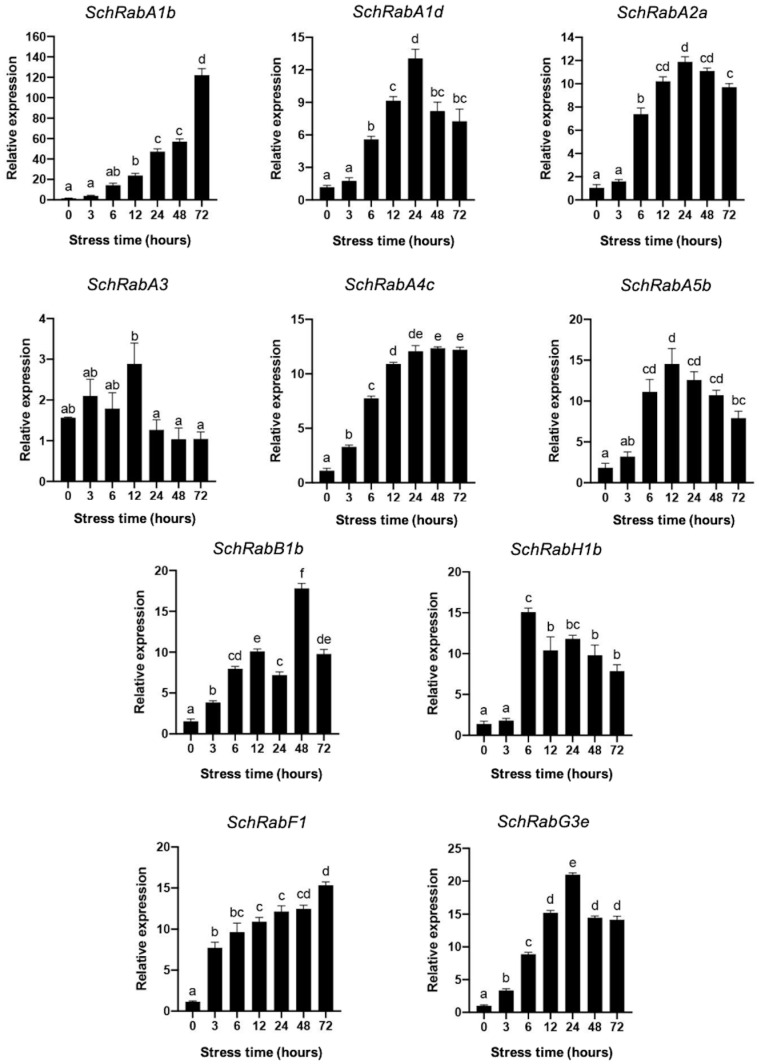
Relative expression of *Rab GTPases* under salt stress in *Solanum chilense* roots. Relative expression of RabA subfamily members from the TGN trafficking pathway, with *SchRabB1b* belonging to the secretory pathway, *SchRabH1b* to the retrograde route, and *SchRabF1* and *SchRabG3e* to the endocytic pathway. Black bars indicate the relative expression of *Rab GTPase* genes in roots. Different letters indicate significant differences (*p* < 0.05) according to a one-way ANOVA test.

The transcript levels of the different *Rab GTPases* were also evaluated in the roots of *S. chilense* plants under salt stress conditions (Figure 8). Unlike in leaves, where only specific genes exhibited increased transcript levels, all the genes in roots showed an increase in the transcript levels. However, the different expression levels and variations in induction times persisted among genes within the same subfamily and across different subfamilies. In this sense, *SchRabA1b* and to a lesser extent *SchRabG3e* stand out for their significantly high levels of induction. On the other hand, not all genes maintained high levels of expression during the 72 h of salt treatment. Interestingly, and unlike what was observed in leaves, *SchRabA3* expression seemed to be repressed after 12 h of treatment.

## 4. Discussion

Rab GTPases are important proteins in vesicle transport, being involved in the whole process of vesicle generation, docking, binding, and fusion with the target membrane [10,38,39]. The Rab GTPase family is highly conserved in different organisms, such as yeast, mammals, and plants [28,29,40], thus providing the basis for vesicle transport in an organism.

### 4.1. The Rab GTPase Superfamily Is Represented in S. lycopersicum with 54 Members

In this work, 54 putative non-redundant Rab GTPases were identified in the genome of *S. lycopersicum*, three members less than in the genome of *A. thaliana* [28]. The Rab GTPases found in the genome of *S. lycopersicum*, as in *A. thaliana*, are divided into eight subfamilies. This fact is consistent with observations in other organisms such as mammals, yeast, mango, rice, maize, and poplar [26,40,41,42,43]. Rab GTPases are phylogenetically grouped according to their subfamily, indicating that their members share functions and locations within the cell [40]. The largest family in *S. lycopersicum* is RabA, which is consistent with the expansion of this subfamily in plants [28,29]. Rab GTPases, being highly conserved among organisms, maintain a size close to 200 amino acids, with an average weight of 24 kDa (Table 1), coinciding with the average weight described for Rab GTPases [44]. There are only two exceptions. Solyc11g012460, which corresponds to the largest putative Rab GTPase (30 kDa, 277 amino acids), differs from its *A. thaliana* homolog (*AT5G47520*, corresponding to *AtRabA5a*) at the C-terminal end of the nucleotide sequence, which is longer in the putative Rab GTPase from *S. lycopersicum*. Tomato *RabA5a* has been evaluated as one of the genes that potentially regulates blossom-end rot (BER) [45]. It can be hypothesized that the additional amino acids, when compared to AtRabA, are involved in a response to this disease; however, this needs to be studied further. The second exception in terms of size is Solyc06g005350.2, with only 145 amino acids in its sequence. This sequence is incomplete (Figure 3), since a tomato RabE described by Fleming et al. [46], named Tm3, has 218 amino acids in its sequence, the same number of residues as those found in its *A. thaliana* counterpart and similar to the 217 amino acids found in sequences of the same clade (Figure 1). This sequence, as shown in Figure 1, clusters with RabE of *A. thaliana*, but given its length of the sequence, it cannot be classified specifically with any member of that subfamily but instead is grouped in general with the AtRabE clade.

In the Rab GTPase subfamily B of *S. lycopersicum*, there are five members, while in *A. thaliana* there are only three members. The increase in the number of *Rab GTPases* coincides with some of the gene duplications within the same genome in tomato. The same situation is observed in the case of the RabD subfamily, where the additional *Rab* can be explained by a duplication event.

Intron/exon distribution is maintained depending on the subfamily to which *Rab GTPases* belong, thus maintaining a conserved gene structure. It has been reported that within the Rab GTPase families, there is a tendency to maintain this intron–exon distribution related to a specific function within the same family [40,47,48]. An example is observed in the *Populus trichocarpa* Rab GTPase family, whereas the intron–exon distribution remains conserved within each subfamily (RabA–RabH) [42]. This situation also occurs in other highly sequence-conserved superfamilies, such as the NAC domain transcription factor gene family, where the phylogenetically closest members have a similar intron–exon distribution [49].

The 54 sequences found in the genome of *S. lycopersicum* correspond to Rab GTPases according to the criteria proposed by Pereira-Leal and Seabra [40], with the first condition being the existence of Rab family domains (RabF). These five domains (RabF1–RabF5) characterize a Rab GTPase. Also crucial is the presence of the so-called hydrophobic triad in the switch region of Rab GTPases, which is key for effector recognition [34,35]. This hydrophobic triad is present in all 54 sequences of putative Rab GTPases (Figure 4). These residues, also known as the aromatic triad, correspond to phenylalanine, tryptophan, and a variable tyrosine/phenylalanine residue [36]. Against this background, it is possible to indicate that the sequences found in the genome of *S. lycopersicum* correspond to Rab GTPases.

### 4.2. High Functional Diversity of Rab GTPases in Solanaceae

The transcriptional analysis carried out in different organs and different stages of plant development of *S. lycopersicum* and *S. pennellii* (Figure 5) shows that all Rab GTPases are expressed in at least one of the samples analyzed, which suggests that they fulfill specific functions, justifying the high number of genes found in the Solanaceae genome. This observation is common in higher plants, since a similar situation has been described in cotton, soybean, rice, poplar, and even in Arabidopsis [28,29]. On the other hand, some genes show conserved expression patterns within the genus, such as the three RabGs, some RabAs and the three RabHs. This is interesting, since it suggests that the trafficking routes in which they are involved are vital for the development of the plant and consequently must be maintained or conserved. Therefore, it seemed appropriate to analyze the transcriptional profile of other salt-tolerant species related to tomato (Figure 6), in order to know the specific Rab GTPases that could have evolved due to selection pressure induced by unfavorable environmental conditions. Of the three wild species analyzed, *S. pennellii* has the greatest tolerance, and *S. pimpinellifolium* has the lowest tolerance (Figure 6). Here, the results show that the transcriptional pattern increases as the species becomes more tolerant. This situation is observed for the genes belonging to the RabB and RabD subfamilies, which suggests that, indeed, the regulation of the expression of these genes is in the process of evolution, which coincides with what was described by Pereira-Leal et al., 2001 [40].

### 4.3. Differential Expression of Rab GTPases of the Endocytic and TGN Pathway in S. chilense under Stress Conditions

Of the total Rab GTPases found in the genome of *S. lycopersicum*, ten genes were selected to be evaluated under salt stress conditions in *S. chilense* plants. This halophyte species can grow in the Atacama Desert in northern Chile, one of the driest areas in the world. It simultaneously tolerates conditions of salinity, drought, and extreme temperatures [50,51]. In salt stress conditions, *S. chilense* showed a higher accumulation of ROS detoxifying antioxidant enzymes than *S. lycopersicum*, allowing continual operation of the cellular machinery in normalized conditions under such stress [52].

Expression profiles were obtained in both leaves and roots for each of these ten genes in *S. chilense*. It is interesting to note the high levels of induction of genes belonging to the RabA1 subfamily (*SchRabA1b* and *SchRabA1d*) under salt stress conditions, occurring at both root and leaf levels (Figure 7 and Figure 8). These results are consistent with the observations of Asaoka et al. [15], who determined that RabA1a-d Rab GTPases are required and necessary for salt stress tolerance, regulating the localization of cell surface proteins, such as channels and pumps. Being a highly conserved superfamily between species [28,40,53], it has been reported that the function of each of its members is maintained even in different organisms, so it could be assumed that the function of these two Rab GTPases (SchRabA1b and SchRabA1d) is of great importance under salt stress, explaining the important transcriptional induction of these genes under this type of stress.

*SchRabA3* shows a different behavior than other Rab GTPases, increasing its transcript levels at 12 h, where it reaches the peak; however, after this point there is a decrease in transcript levels to even lower levels than those presented at time 0. This *Rab* was amplified using primers obtained from the sequence of *Solyc11g012460.1*, which is the sequence with the largest size among the *S. lycopersicum Rab GTPases*. Since *S. lycopersicum* and *S. chilense* are close relatives, the nucleotide sequence could have high similarity and therefore related functions. The *Rab* of *S. lycopersicum Solyc11g012460.1* has a role in biotic stress [45] and has also been reported to contribute to fruit firmness in *M. indica* [54]. This gene also showed an interesting behavior in seedling roots in *S. lycopersicum* and *S. pennellii*, being one of the few genes to exhibit negative expression levels in such an organ (Figure 5).

Regarding *SchRabH1b*, whose expression in leaves is repressed compared to the control, there are precedents in maize crosses between resistant and tolerant varieties, where repression of this gene is observed when subjected to salt stress conditions. The authors speculated that this protein can be a salt stress-negative regulatory protein [55]. However, in roots of *S. chilense*, its expression is strongly induced starting 6 h after applying the stress, indicating that it could be fulfilling a different function in leaves than in roots.

Rab GTPases of the endocytic pathway have been shown to play an important role in salt stress tolerance. There is induction of genes representing this pathway when subjected to stress conditions, as is the case of *SchRabF1* (Solyc11g008430) and *SchRabG3e* (Solyc01g109520) in leaves (Figure 7) and roots (Figure 8). There is evidence that members of the RabF subfamily play a role in salt stress tolerance, such as the mutation that increases AtRab ARA7 (AtRabF2b) protein levels, which resulted in increased long-term tolerance to salt stress when compared to WT plants. In addition, an increased mass of roots and leaves, increased K^+^ content, and decreased Na^+^ content have been reported under these conditions [14]. Moreover, AtRabF1 of *A. thaliana* transports endosomal vesicles and plays a role in stress response. Overexpression of this gene both in its activated and inactivated state increases stress tolerance when compared to WT plants, exhibiting longer roots and playing a role in dark-induced senescence [25]. Additionally, *SchRabF1* shows higher expression levels in *S. pennellii* seedling roots when compared to *S. lycopersicum* (Figure 5). Considering this background, it is possible to infer that *SchRabF1* could be relevant in future studies of salt stress tolerance, given its induction in expression levels in both leaves and roots.

The expression in *S. chilense* of *SchRabG3e*, a *Rab GTPase* involved in the endocytic pathway of intracellular vesicular trafficking, is highly transcriptionally induced under salt stress conditions. This gene also shows higher expression levels in roots of *S. pennellii* seedlings when compared to *S. lycopersicum* (Figure 5). The role of different Rab GTPases of the RabG subfamily involved in abiotic stress tolerance processes has been described. The role of *AtRabG3e* in salt stress tolerance was previously demonstrated in *A. thaliana* [56], where better germination and robust growth were observed in plants overexpressing this gene. Furthermore, overexpression of *PgRab7* from *P. glaucum* or *PjRab7* from *Prosopis juliflora*, which are homologs of *RabG3e* from *A. thaliana*, confers salt stress tolerance in transgenic tobacco plants [27]. Similarly, overexpression of *OsRab7* in rice increased the tolerance of rice plants to drought and heat [57]. Considering this evidence, it is also possible to infer that *SchRabG3e* could be used in future studies of salt stress tolerance, given its high expression levels in both leaves and roots of tomato plants.

## 5. Conclusions

The Rab GTPase family has been characterized in other species previously, but to our knowledge, no systematic study has been performed in *S. lycopersicum*. We found 54 putative Rab GTPases in the genome, which are distributed in eight subfamilies (RabA–RabH) as described in the literature. Along with this, the sequences show a high degree of conservation, maintaining the size and weight at the amino acid level, the characteristic motifs of this family, and a conserved number of introns within each subfamily.

Once the sequences coding for Rab GTPases in *S. lycopersicum* were obtained, the expression profiles of some of these were determined in *S. chilense*, where their expression profiles, together with data obtained from databases, account for the transcriptional activation that occurs in wild species as well as under salt stress conditions. The profiles of *SchRabA1b* and *SchRabG3e* under stress conditions are particularly noteworthy, establishing them as candidate genes for future studies.

## Figures and Tables

**Table 1:** Putative Rab GTPases in the genome of *Solanum lycopersicum*. Chromosome location, genome coordinates (StartPos–EndPos), strand orientation (“+”: direct orientation, “-”: indirect orientation), protein length in amino acids, isoelectric point (pI) molecular weight (Mw) in kDa, GRAVY (Grand Average of Hydropathicity), and subcellular location are indicated. Subcellular locations are abbreviated as follows: Cyt: Cytoplasmic; Chl: Chloroplast; Nuc: Nuclear; Gol: Golgi complex; Csk: Cytoskeleton; Ex: Extracellular.

GeneName	ChrName	Chromosome Location			Length (aa)	Isoelectric Point	Mw (kDa)	GRAVY	Subcellular Location	
		Start	End	Strand					WPS	CELLO
Solyc01g086850.2	1	81,771,059	81,775,731	-	226	6.76	24.78	−0.192	Cyt	Cyt
Solyc01g088560.2	1	83,336,020	83,341,655	-	209	7.67	23.01	−0.162	Chl	Chl
Solyc01g090170.2	1	83,802,202	83,809,193	+	204	5.1	22.45	−0.254	Cyt	Gol
Solyc01g096220.2	1	87,314,716	87,316,882	+	244	6	27.1	−0.199	Nuc	Cyt
Solyc01g103370.2	1	91,989,852	91,994,839	+	203	5.33	22.5	−0.277	Nuc	Cyt
Solyc01g103380.2	1	91,995,157	91,998,149	-	215	6.22	23.8	−0.291	Chl	Chl
Solyc01g109520.2	1	96,449,221	96,452,841	-	207	5.1	23.01	−0.397	Chl	Chl
Solyc02g036450.2	2	30,788,590	30,794,213	-	201	7.75	21.96	−0.181	Chl	Chl
Solyc02g069370.2	2	39,281,375	39,289,536	-	201	5.89	21.66	−0.155	Chl	Chl
Solyc02g072180.2	2	41,496,822	41,503,133	+	218	5.5	24.09	−0.375	Nuc	Cyt
Solyc02g081380.2	2	45,368,874	45,373,407	-	201	6.15	21.82	−0.216	Chl	Chl
Solyc02g093530.2	2	54,369,660	54,374,256	-	212	7.72	23.29	−0.293	Cyt	Cyt
Solyc03g006270.2	3	888,904	890,977	+	217	5.63	24.01	−0.290	Cyt	Cyt
Solyc03g064020.2	3	37,223,959	37,225,122	+	225	6.12	24.96	−0.204	Cyt	Cyt
Solyc03g078570.2	3	51,178,678	51,181,240	-	208	6.39	23.14	−0.255	Chl	Cyt
Solyc03g079900.2	3	51,787,671	51,789,397	+	216	6.97	23.96	−0.264	Csk	Cyt
Solyc03g118820.2	3	67,626,322	67,629,418	+	243	5.37	23.16	−0.301	Nuc	Cyt
Solyc03g120750.2	3	69,027,066	69,031,274	-	206	5.21	22.97	−0.338	Chl	Cyt
Solyc04g011360.2	4	3,839,267	3,844,110	-	217	7.65	23.9	−0.378	Nuc	Chl
Solyc04g012180.2	4	4,461,532	4,467,418	-	223	8.44	24.64	−0.266	Cyt	Cyt
Solyc04g051680.2	4	50,982,372	50,985,730	-	220	4.93	24.71	−0.284	Cyt	Cyt
Solyc04g064510.2	4	55,670,912	55,675,897	-	210	5.4	23.27	−0.212	Chl	Chl
Solyc04g072060.2	4	59,116,633	59,126,825	-	205	5.46	22.93	−0.319	Chl	Cyt
Solyc05g051570.2	5	61,938,794	61,942,841	-	208	6.4	23.09	−0.169	Chl	Chl
Solyc05g052070.2	5	62,410,368	62,413,664	-	204	5.27	22.62	−0.373	Cyt	Cyt
Solyc05g052900.2	5	63,076,702	63,078,931	-	216	7.68	23.99	−0.240	Cyt	Cyt
Solyc05g053940.2	5	63,958,473	63,963,310	-	204	5.26	22.41	−0.264	Csk	Gol
Solyc05g054150.2	5	64,106,692	64,108,791	+	227	5.84	25.05	−0.388	Cyt	Cyt
Solyc06g005350.2	6	362,156	370,204	-	145	9.44	16.14	−0.187	Cyt	Chl
Solyc06g005810.2	6	839,934	842,843	-	217	6.1	23.83	−0.289	Cyt	Cyt
Solyc06g060210.1	6	38,174,983	38,175,594	+	204	8.8	23.06	−0.357	Chl	Cyt
Solyc06g076450.2	6	47,498,298	47,500,384	-	216	6.44	23.82	−0.244	Cyt	Cyt
Solyc07g053480.2	7	61,927,834	61,930,515	+	218	5.66	24.09	−0.254	Cyt	Cyt
Solyc07g055290.2	7	63,373,504	63,378,793	-	219	5.73	24.16	−0.316	Cyt	Cyt
Solyc07g056150.2	7	63,994,885	63,999,176	-	213	6.9	23.19	−0.194	Cyt	Mit
Solyc08g078070.2	8	61,918,544	6,1921,746	+	204	5.48	22.5	−0.302	Nuc	Gol
Solyc09g008460.2	9	1,905,656	1,911,197	-	217	5.73	23.65	−0.274	Chl	Chl
Solyc09g010370.2	9	3,753,893	3,759,018	-	217	7.65	23.95	−0.361	Chl	Chl
Solyc09g056340.2	9	48,781,284	48,783,613	+	211	4.97	23.61	−0.353	Cyt	Cyt
Solyc09g097900.2	9	71,891,302	71,896,403	-	217	4.96	24.26	−0.337	Cyt	Cyt
Solyc09g098170.2	9	72,044,889	72,048,527	+	218	5.13	24.29	−0.410	Cyt	Cyt
Solyc10g007700.2	10	1,953,681	1,958,548	-	212	6.9	23.12	−0.221	Cyt	Cyt
Solyc10g008840.2	10	2,900,438	2,901,941	+	216	5.53	24.29	−0.256	Cyt	Cyt
Solyc10g045550.1	10	34,584,594	34,590,416	+	216	8.34	23.92	−0.287	Cyt	Chl
Solyc10g079030.1	10	60,670,713	60,672,194	-	227	6.32	24.94	−0.200	Chl	Mit
Solyc10g086350.1	10	65,205,566	65,210,133	-	217	8.37	23.87	−0.349	Chl	Chl
Solyc11g008020.1	11	2,238,213	2,241,626	+	217	9.28	24.26	−0.319	Chl	Ex
Solyc11g008430.1	11	2,614,324	2,620,969	-	201	5.91	21.57	−0.191	Cyt	Chl
Solyc11g010100.1	11	3,222,459	3,224,358	+	225	6.33	24.57	−0.199	Cyt	Cyt
Solyc11g012460.1	11	5,297,608	5,298,810	+	277	5.31	30.85	−0.383	Nuc	Nuc
Solyc11g073050.1	11	56,164,984	56,168,738	+	217	8.37	23.8	−0.313	Chl	Chl
Solyc12g010790.1	12	3,711,266	3,713,629	-	218	5.48	24.05	−0.253	Cyt	Cyt
Solyc12g011130.1	12	3,971,227	3,974,056	-	212	6.9	23.14	−0.212	Cyt	Cyt
Solyc12g014080.1	12	4,894,053	4,896,737	-	218	5.53	24.29	−0.256	Cyt	Cyt

**Table 2:** *Rab GTPases* homologous of *Solanum lycopersicum* in *Solanum chilense*. For the *S. chilense* Rab GTPase sequences used for differential expression assays under salt stress conditions, the names of those homologous *S. lycopersicum* Rab GTPases were used. Then, based on the phylogenetic tree and given the high conservation in this superfamily, they were renamed according to the nomenclature used in *A. thaliana*.

Homologous Sequence in *S. lycopersicum*	Name Given to Rab GTPases in *S. chilense* (Reference to Arabidopsis Rab GTPases)
Solyc07g055290	SchRabA1b
Solyc02g072180	SchRabA1d
Solyc01g103380	SchRabA2a
Solyc01g096220	SchRabA3
Solyc01g086850	SchRabA4c
Solyc09g056340	SchRabA5b
Solyc02g093530	SchRabB1b
Solyc01g088560	SchRabH1b
Solyc11g008430	SchRabF1
Solyc01g109520	SchRabG3e

## Data Availability

Data are contained within the article and Supplementary Materials.

## Supplementary Materials

The following supporting information can be downloaded at: https://www.mdpi.com/article/10.3390/genes15040453/s1, Figure S1: Physiological parameters and relative expression levels of stress marker gene *TSW12*; Table S1: Nucleotide sequence of the primers.
