# Supplementary figures and images for: Identification, Classification, and Transcriptional Analysis of Rab GTPase Genes from Tomato (Solanum lycopersicum) Reveals Salt Stress Response Genes
Source: Genes (Basel). 2024 Apr 3;15(4):453. doi: 10.3390/genes15040453 (PMC11049601; doi:10.3390/genes15040453)

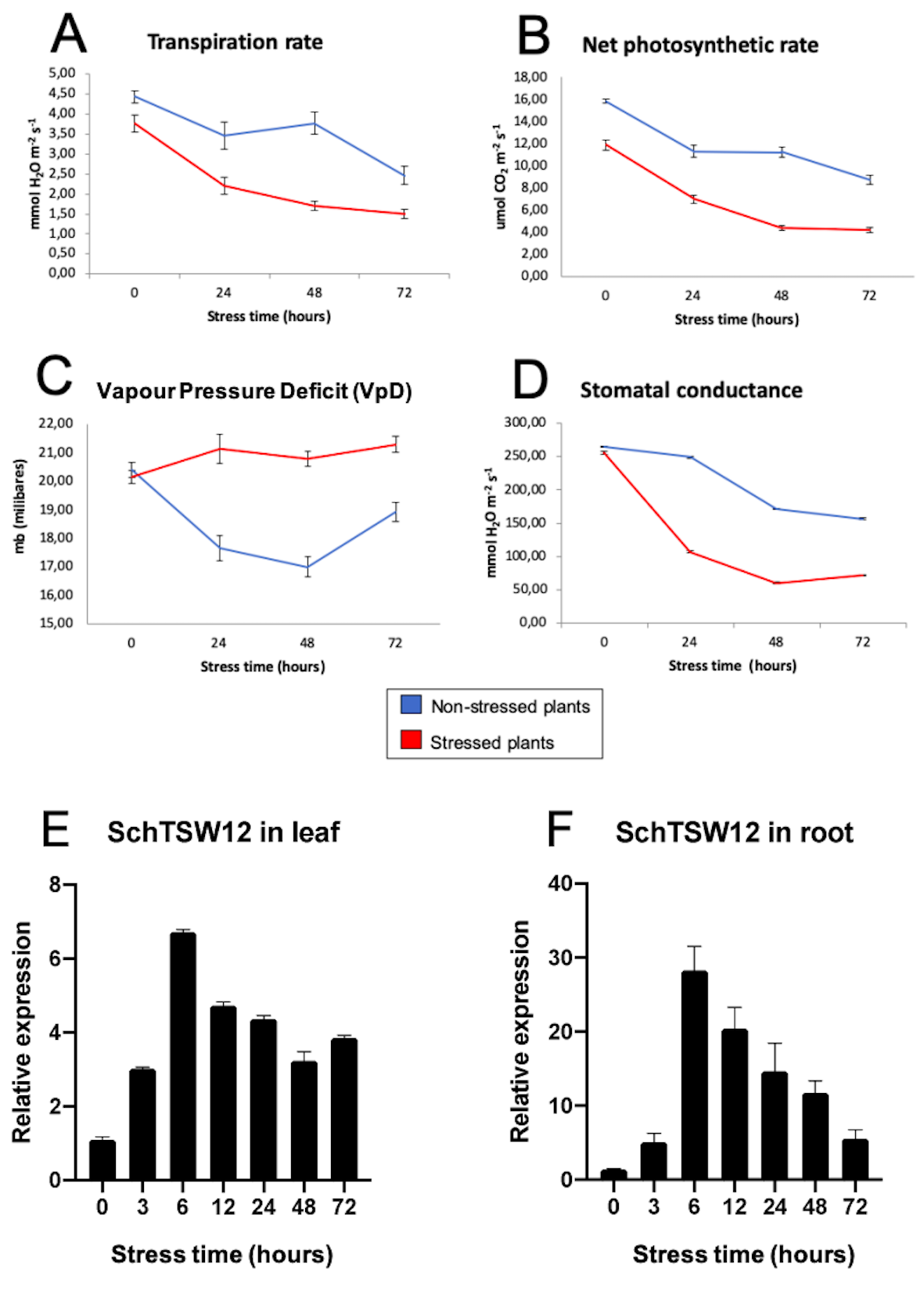

Supplement: Supplementary file 1 [file genes-15-00453-s001.zip › Supplementary Materials/Figure S1.png]
